# Case Report: A tortuous diagnosis and successful multimodal treatment of thyroid follicular carcinoma with pelvic metastasis

**DOI:** 10.3389/fonc.2023.1048485

**Published:** 2023-05-19

**Authors:** Zhi-Liang Hong, Hai-Jian Huang, Sheng Chen, Jian-Chuan Yang, Song-Song Wu

**Affiliations:** ^1^ Shengli Clinical Medical College, Fujian Medical University, Fuzhou, China; ^2^ Department of Ultrasound, Fujian Provincial Hospital, Fuzhou, China; ^3^ Department of Pathology, Fujian Provencal Hospital, Fuzhou, China

**Keywords:** pelvic metastasis, MDT, ultrasound, multimodal treatment, thyroid follicular carcinoma

## Abstract

**Purpose:**

To provide reference method for the treatment of thyroid follicular carcinoma by studing the clinical imaging, pathological features and multimodal treatment of a case of thyroid follicular carcinoma with bone metastasis.

**Methods:**

By identifying the case’s clinical, imaging, pathological features of a case of thyroid follicular carcinoma with bone metastasis, reflecting on the case’s diagnosis and treatment process, and referring to literature about the characteristics of thyroid follicular carcinoma, the study aims to provide reference for the treatment of this kind of disease.

**Result:**

A 67-year-old male patient was admitted to the hospital with clinical symptoms of left pelvic pain. The biopsy pathology showed well-differentiated thyroid tissue. Considering his medical history, conclusion of thyroid follicular carcinoma metastasis could be made.The patient was stable and no tumor progression was observed after a combination of therapies including ^131^I and topical and targeted agents.

**Conclusions:**

Thyroid follicular carcinoma are prone to bone metastasis, and bone metastasis is the first symptom in some cases. Clinical imaging and pathology are needed for correct diagnosis, and a successful treatment requires a combination of multiple approaches including ^131^I, which is a Radioactive Iodine Therapy(RAI), local therapy and targeted drug therapy.

## Case presentation

**Figure 1:**
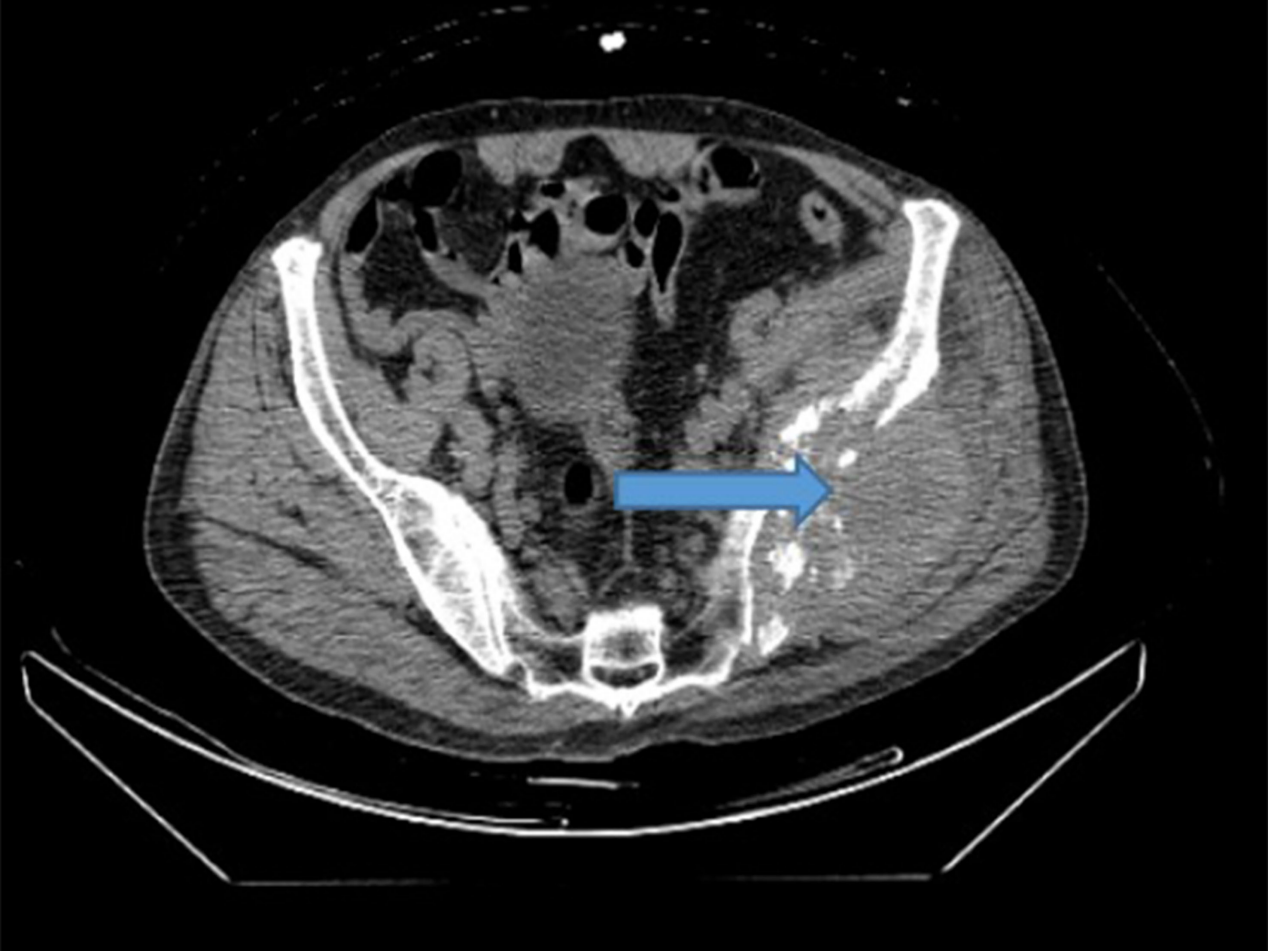
Preoperative SPECT/CT showed bone destruction in the corresponding part of the left iliac crest and soft tissue mass in the left iliac crest lesion, which was considered as bone metastasis.

**Figure 2:**
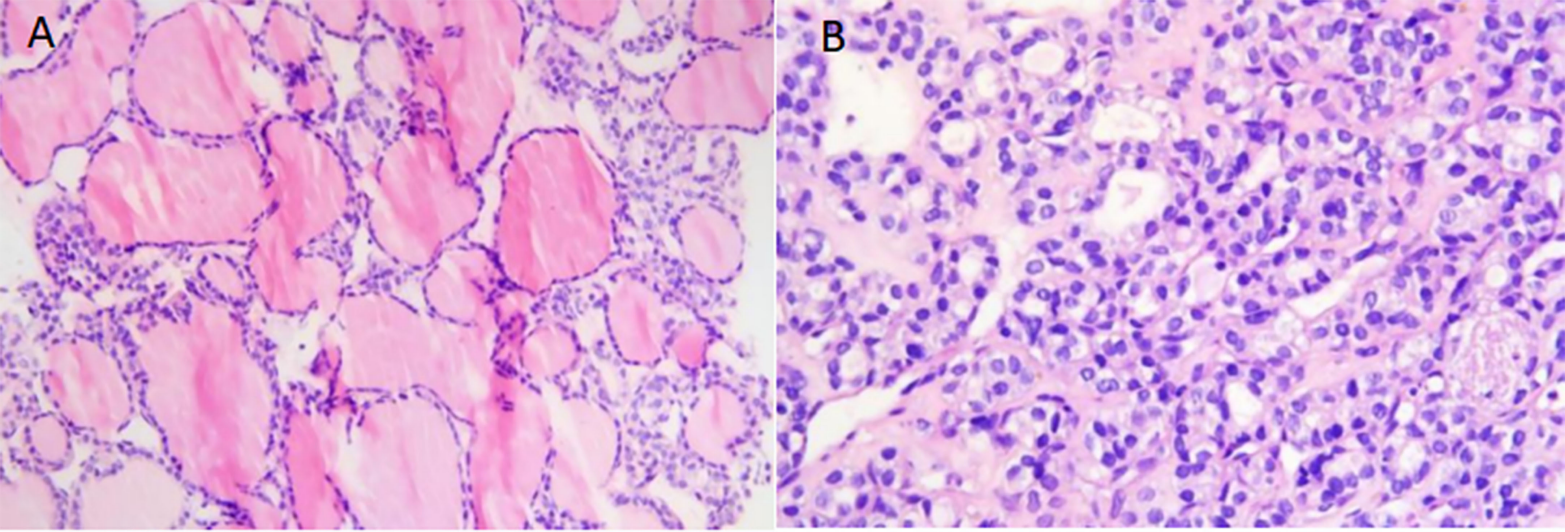
**(A)** Microscopically, thyroid follicles of different sizes were observed, and the cells were well differentiated. No ground-hyaline nuclei, nuclear furrows, sand bodies, or papilla were observed. The appearance of follicular tumor was consistent with bone metastasis of thyroid follicular carcinoma. (original magnification, × 400; hematoxylin-eosin [H-E] stain). **(B)** The tumor cells were dense and crowded, similar to fetal thyroid tissue, with a small number of follicles, nuclear atypia, and rare pathological mitosis.(original magnification, × 600; hematoxylin-eosin [H-E] stain).

**Figure 3:**
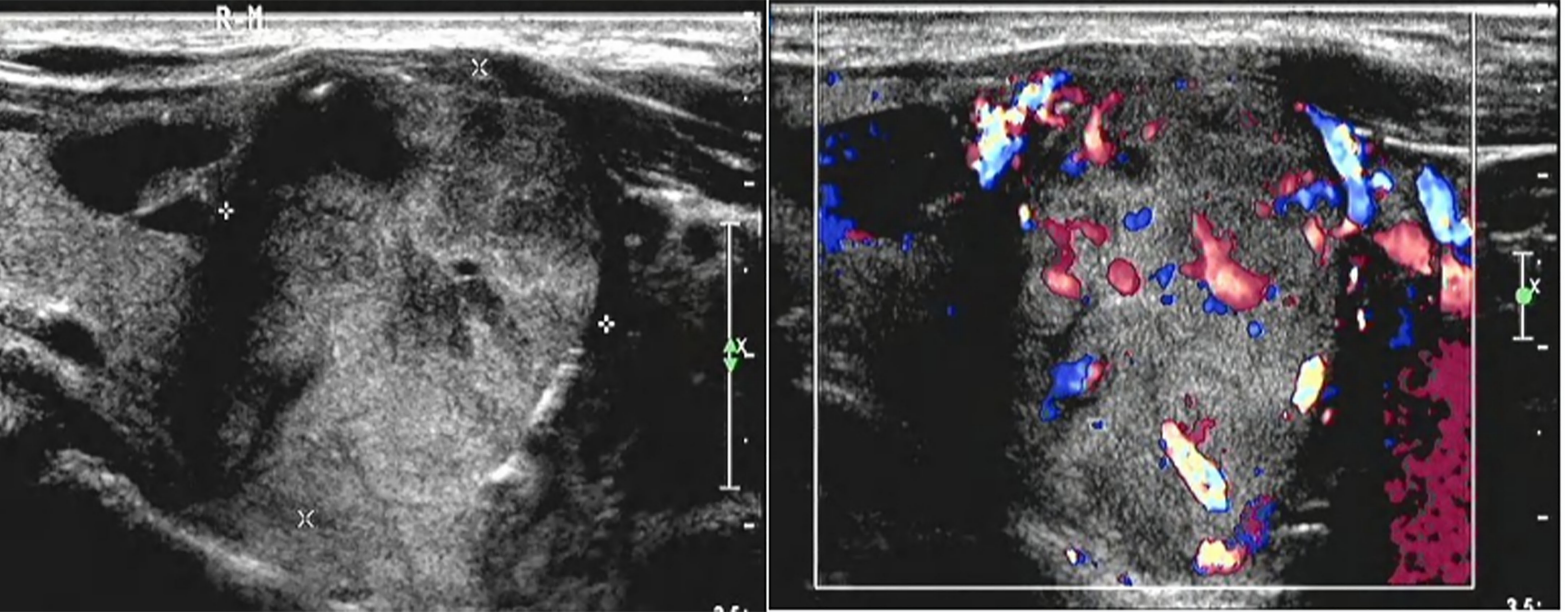
There was a solid lobe nodule on the right side of the thyroid gland, about 30mm × 28mm in size, with clear boundary, mainly solid in the thyroid gland, with irregular and non-echogenic inclusions. A short strip of strong echo could be seen around the nodule, and rich blood flow signals could be seen in and around the nodule.

**Figure 4:**
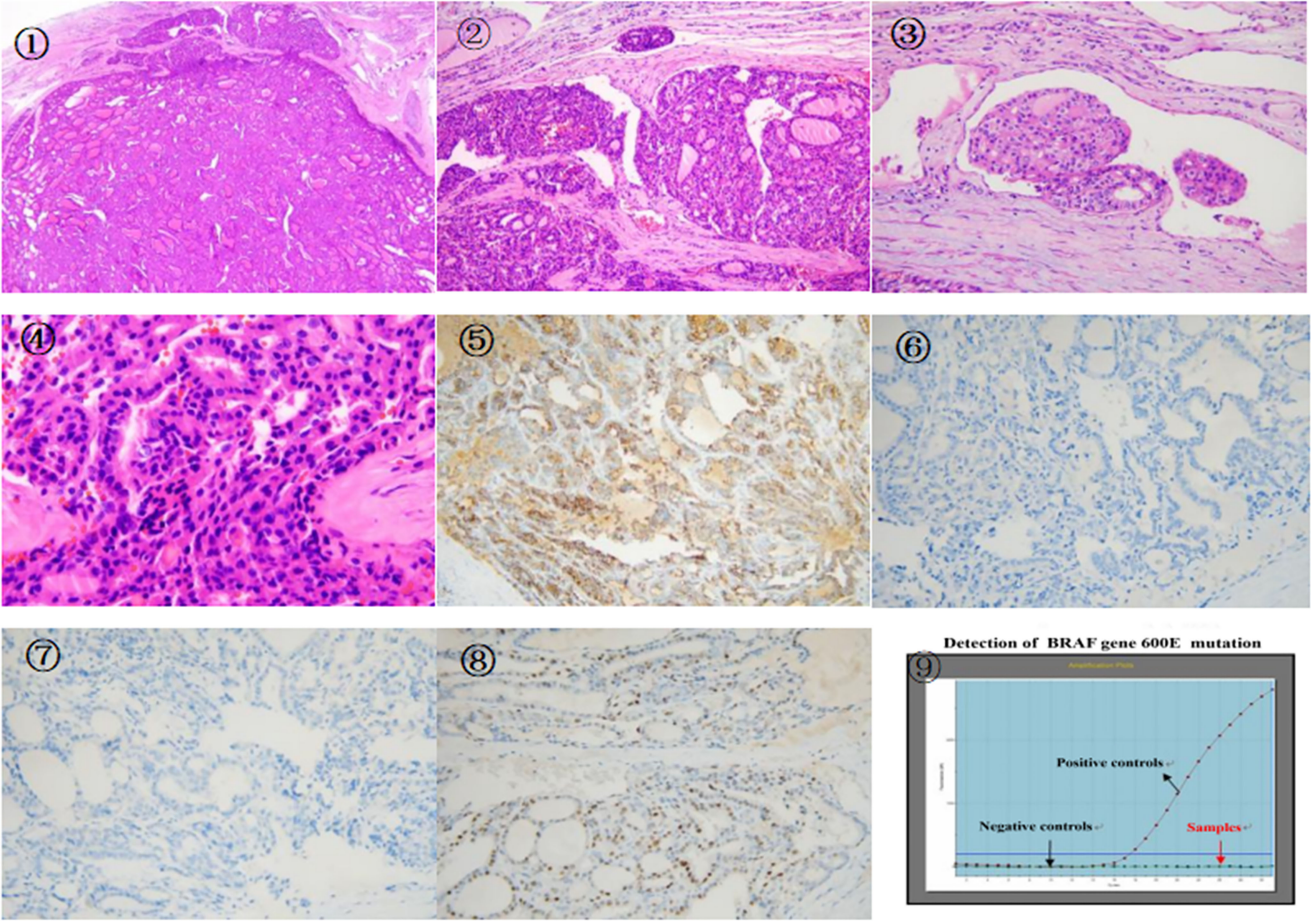
①There is a follicular tumor, fibrous capsule was seen around the tumor,the tumor invaded the capsule and vasculature,extrapulocapsular and intravascular tumor thrombus can be seen.(original magnification, × 20; hematoxylin-eosin [H-E] stain). ② An enlargement of the previous picture,the tumor invaded the vasculature and capsule,there was a vascular tumor thrombus.tumor cells had eosinophilic cytoplasm,the cells showed mild atypia,(original magnification, × 100; hematoxylin-eosin [H-E] stain). ③ The tumor invaded the vasculars and tumor thrombus,(original magnification, × 400; hematoxylin-eosin [H-E] stain). ④ High magnification of tumor invasion capsule,the tumor cells were dense and crowded, the nuclei were hyperchromatic, there was no ground hyaline nucleus, no nuclear furrow, no nuclear overlap, and no sand body (original magnification, × 600; hematoxylin-eosin [H-E] stain). ⑤ Some cells of MC were positive by immunohistochemistry EliVision method magnification at medium power. ⑥ Immunohistochemistry: Tumor cells were negative for CK19 EliVision method magnification at medium power. ⑦ Immunohistochemistry showed that the tumor cells were negative for Galectin-3 EliVision method magnification at medium power. ⑧ Immunohistochemistry showed that a small number of tumor cells were positive for CyclinD1 EliVision method magnification at medium power. ⑨ BRAF V600E gene mutation was not detected by RTPCR.

A 67-year-old male patient developed left pelvic pain without obvious cause in January 2017. The patient went to hospital, and yet no obvious abnormality was found in the anterolateral radiographs of the pelvis. The doctor in that hospital suggested further examination to exclude the possibility of lumbar disc herniation, but the patient did not follow his advice. In March 2018, the pain in the left pelvis and left lower limb aggravated so much as to cripple the patient. Magnetic resonance imaging (MRI) in other hospitals suggested that the left pelvis was occupied, and malignant tumor was considered. To check out the diagnosis and seek treatment, he went to our hospital in March 2019. SPECT/CT imaging of the whole body (**Figure 1**) suggested: 1) abnormal concentration of imaging agent was distributed in the left iliac bone and the left part of T12 vertebrae, and bone destruction was observed in the corresponding parts; 2) abnormal concentration of imaging agent was observed in the left iliac crest near the symphysis pubis, lateral to the 8th spine of the right posterior rib, and in the lower segment of the right humerus; there is possibility of bone metastasis tumor, which need further diagnosis. Ultrasound-guided puncture biopsy of the left iliac crest was thus sent for pathology analysis. Under microscope, thyroid follicles of different sizes were observed, with mild atypia and mild morphology (**Figure 2A**); dense and crowded glands with active hyperplasia and mild atypia were observed, with a few ground-glass nuclei seen and no nuclear sulcus, no sand bodies, and no papilla observed (**Figure 2B**). Bone metastasis of thyroid follicular carcinoma was thus considered. Reviewing the patient’s past medical history, we found the patient admitted to our hospital 5 years ago due to right neck mass. He underwent thyroid doppler ultrasound examination (**Figure 3**), which showed: There was a solid nodule in the right lobe of the thyroid gland, about 30mm×28mm in size, with clear boundary, solid interior and irregular anechoic area; short strip-shaped strong echo could be seen around the nodules, and abundant blood flow signals could be seen in and around the nodules. Clinical conclusion suggested the strong possibility of nodular goiter accompanied by adenomatoid hyperplasia, and the to-be-determined possibility of follicular tumor. Total thyroidectomy on the right side + subtotal thyroidectomy on the left side + lymph node dissection on the right side of the neck were thus performed. The pathological diagnosis showed the nodule to be adenoma, which did not suggest malignancy. Immunohistochemistry: Ki67 (1%+), Galectin-3(+), MC (+ +), CK19 (+ +). Molecular pathology: no mutation of BRAF gene V600E was detected. Nodular goiter (left thyroid gland) with adenomatous hyperplasia, accompanied by cystic changes. Clinical considerations: The right thyroid mass 3 years ago may be follicular carcinoma (pathologically misdiagnosed as a benign nodule), and this bone metastasis was caused by the gradual increase of hemorrhagic metastasis. After MDT consultation, it was deemed necessary to further resect the left residual thyroid gland. Therefore, the patient was transferred to the Department of Tumor Surgery of our hospital for left residual thyroid lobectomy. Postoperative pathology: nodular goitre (left thyroid lobule), no signs of malignant tumor. To further confirm the diagnosis, the original pathology of the patient’s right thyroid in 2014 was reviewed (**Figure 4**). It said: At low magnification, the tumor was characterized by a follicular neoplasm with a thick fibrous capsule surrounding it; thyroid follicles of different sizes can be seen in the capsule; some thyroid follicles were similar to fetal thyroid follicles during embryonic development; the cells were densely crowded, thyroid glia was found in the follicles, some glia were concentrated, and absorption vacuoles were formed in the follicular epithelium; Right in the upper side of the view, the tumor was seen to invade the capsule and protrude out of it, creating what was called a “mushroom-like appearance”. The tumor invaded the vasculature and formed intracavascular tumor thrombus. At high magnification, the nuclei of the tumor were found slightly enlarged; nucleoli were seen in a few tumor cells; the nuclei were sparse; no ground-glass nuclei, no nuclear groove, no nuclear overlap, and no mitotic images were observed; in some areas, the cytoplasm of tumor cells was eosinophilic; fibrous hyperplasia was seen in the tumor stroma and separated the tumor into nodular structures; the surrounding thyroid tissue was nodular goiter. Based on two thyroid surgeries, imaging tests, and the histopathological characteristics of thyroid follicular carcinoma, the final diagnosis of right thyroid follicular carcinoma with pelvic metastasis was made.

**Figure 5:**
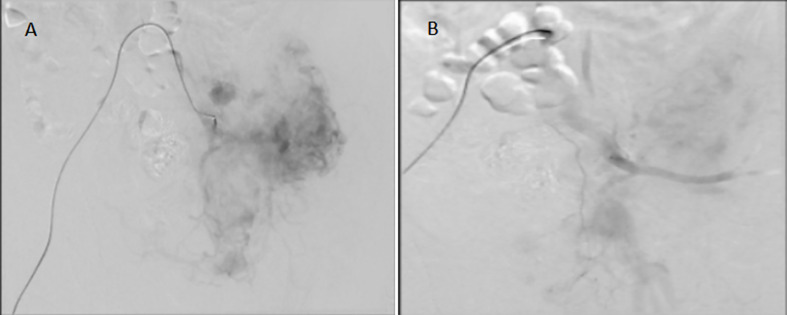
Embolization of the left pelvic tumor artery. **(A)**: before tumor artery embolization; **(B)**:after tumor artery embolization.

**Figure 6:**
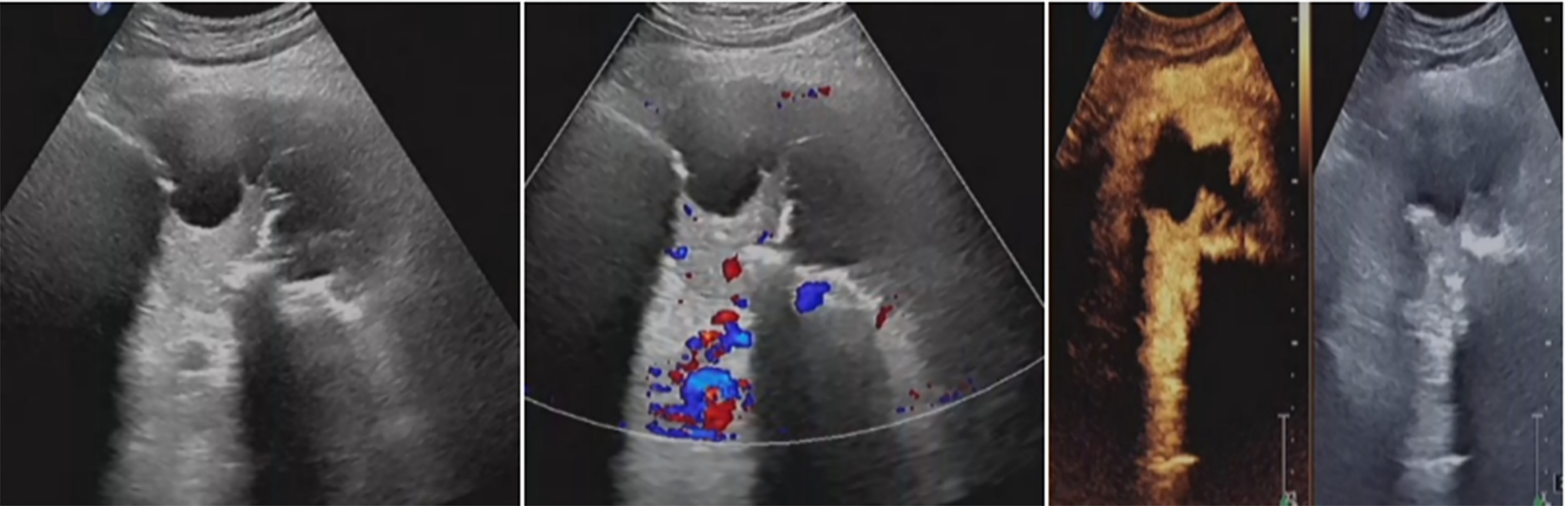
Residual tumor was inactivated by ultrasound-guided fractionated radiofrequency ablation. The ablation range was preliminarily estimated to reach 90%, achieving the expected therapeutic goal.

**Figure 7:**
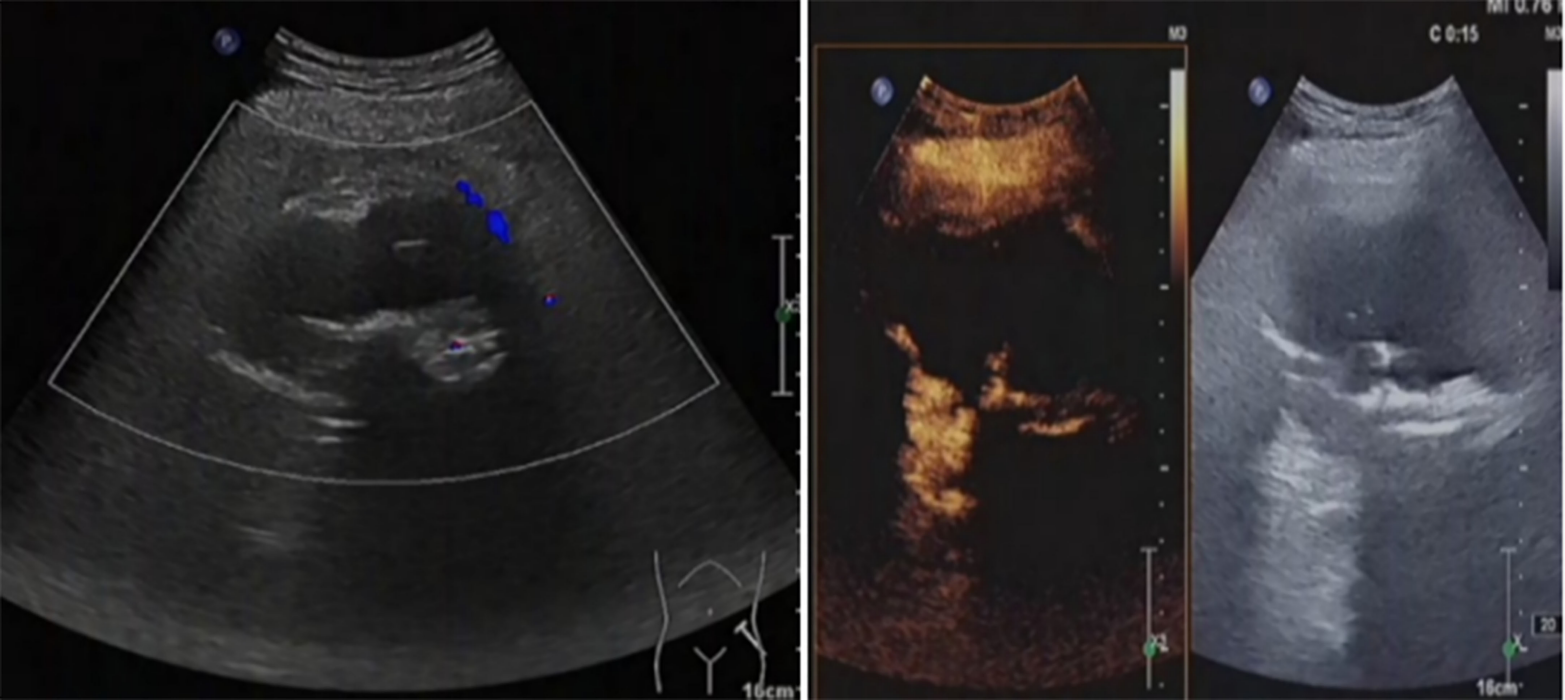
The hip tumor lesion was reduced compared with that before surgery, with a range of about 83mm × 54mm. No blood flow signal was observed in most areas, and the strip artery blood flow signal was observed in the medial margin, indicating no obvious activity in the lesion.

**Figure 8:**
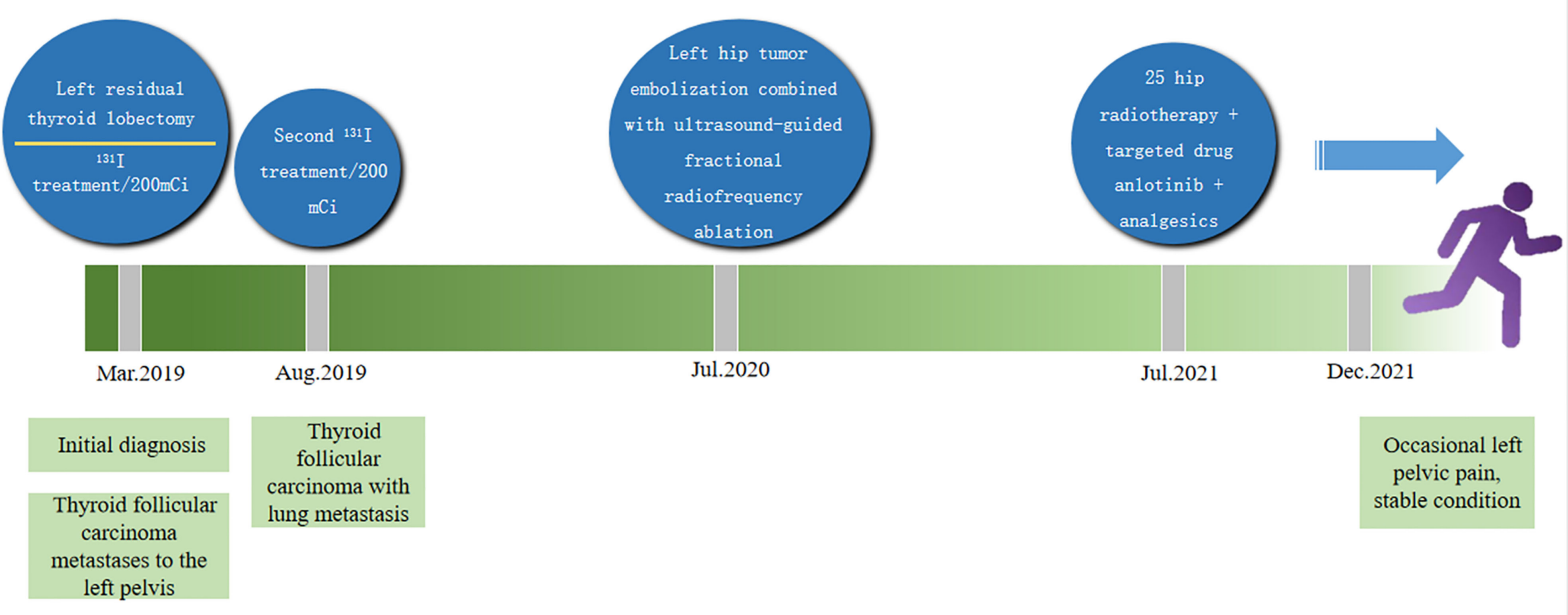
The treatment of large pelvic metastases which was successfully controlled by multiple methods.

The subsequent treatment course was as follows:^131^I treatment which is a Radioactive Iodine Therapy(RAI), was performed in the Nuclear Medicine Department of our hospital with a dose of 200mCi. Two weeks after surgery, the patient reported relief of pain in the left pelvis. After a few months of rest at home, the patient underwent a whole-body bone SPECT/CT scan in August 2019, which revealed multiple miliary and nodular thickened shadows in both lungs and indicated the possible lung metastasis of thyroid follicular carcinoma. No obvious change was found with the bone metastasis. The second ^131^I treatment was then performed in the Nuclear Medicine Department of our hospital with a dose of 200mCi. The patient complained of pain in the left pelvis and difficulty in walking. After MDT discussion, multi modal sequential combined therapy was proposed: embolization of the blood supply artery to the left pelvic metastasis would be performed in the hope of blocking the blood supply to the main body, followed by fractional percutaneous ablation to inactivate the residual tumor. In July 2020, left pelvic tumor artery embolization was perfomed on the patient in the interventional radiology department of our hospital (**Figure 5**), with left and right internal iliac arteries embolized by gelatin sponge microstrips via catheterization, costing about 120ml of iodoxanol. Angiography findings showed that corresponding to the left iliac bone and adjacent area, angiography of the left internal iliac artery showed tumor vessels; tumor staining was seen in the parenchymal stage; the boundary part was clear; the right internal iliac artery was also involved in tumor blood supply; after gelatin-sponge microstrip embolization, the blood flow of the left and right internal iliac arteries slowed down significantly. Three days after embolization, the patient had no obvious discomfort. Ultrasound-guided fractional radiofrequency ablation (**Figure 6**) was then performed under intravenous basic anesthesia. Intraoperative contrast-enhanced ultrasound showed that the left pelvic metastasis was about 70% inactivated by embolization, with residual active areas seen in the surrounding area. Multi-point and multi-needle radiofrequency ablation was then performed with a power of 120W, which took 30 minutes. Postoperative contrast-enhanced ultrasound showed that the tumor was basically inactivated. However, for fear of serious complications, the medial area close to the left iliac vessel and important nerve passage was left untreated. The operation was successful, and the patient had no obvious discomfort. The second supplementary ablation was performed half a month later. Considering the large local tumor load and multiple lung lesions, radiotherapy and targeted therapy were added. From July 2021, he received 25 rounds of hip radiotherapy successively. At the same time, the patient was treated with 5 courses of targeted drug anlotinib at 12mg qd, each course lasting for two consecutive weeks followed by a 1-weak recovery period. The patient reported abated pain. In December 2021, contrast-enhanced ultrasound (**Figure 7**) was re-examined: The tumor lesion on the left pelvis was smaller than before surgery, with a range of 83mm×54mm, and no blood flow signal was observed in most areas, indicating no significant activity of the lesion. At present, the patient still has occasional pain in the left pelvis, and he takes tramadol, morphine and oxycontin daily for analgesic treatment. Subsequent targeted therapy with anlotinib was performed. The follow-up showed stable condition, with no tumor progression found (**Figure 8**).

## Discussion

In recent years, the incidence of thyroid cancer has gradually increased and became one of the most common malignant tumors. Thyroid follicular carcinoma (FTC) accounted for about 10% of the incidence of thyroid cancer, mainly with hematologic metastasis and prone to distant metastasis. Its invasiveness, metastasis rate and mortality were all higher than papillary thyroid carcinoma (PTC) (1–3), and it was more likely to occur in women over 50 years old (4, 5). FTC often has no obvious clinical symptoms, and thus remains undetected until the patients undergo ultrasound physical examination, or develop related clinical symptoms associated with distant metastasis. The rate of distant metastasis after surgery was 7%-23%, and the sites of metastasis were mainly bone, lung, brain, etc (4, 5). Some studies have shown that 10%-15% of thyroid follicular carcinoma cases can have distant metastasis, the most common sites being lung and bone, followed by brain, liver and skin (6). During the several years after the thyroidectomy, the patient did not receive any treatment because the pathology did not indicate malignancy at that time. The left pelvic mass was not found to be thyroid follicular carcinoma metastasis until the huge metastasis in left pelvis caused pain in left pelvis as well as other symptoms. As a result, the optimal treatment opportunity was missed. Therefore, once thyroid nodules were found and thyroid adenoma or FTC was not excluded in postoperative pathology, sufficient attention must be paid to comprehensive physical examination and improvement of relevant examinations, and close follow-up should be conducted after surgery. When the primary thyroid lesion was highly suspected to be FTC or the patients were suspected of FTC with other organs as the first suspect, they should be advised to undergo whole-body bone imaging, CT, MRI, ultrasound and other related examinations for comprehensive evaluation. If the primary lesion was not diagnosed by the first pathological examination, it was necessary to re-examine the section or histopathological examination of multiple parts of the primary lesion and metastatic lesion should be conducted to avoid missed diagnosis and misdiagnosis. Once pathological diagnosis indicated malignancy, surgical treatment and multidisciplinary treatment were recommended.

Follicular carcinoma of the thyroid shows a variety of morphologic changes histologically, ranging from a well-differentiated follicular structure such as normal thyroid to distinct malignant features. There are a variety of transitional types, so it is sometimes difficult to determine whether it is benign or malignant by cell morphology and histological structure alone. Dinshore proposed that despite the benign histological appearance and relatively long clinical course of primary and metastatic follicular adenoma, the presence of metastasis suggested biological malignancy (7, 8). The patient in our case was diagnosed with follicular thyroid adenoma at the time of goiter resection 6 years ago. Based on the above point of view, we believe that the thyroid tumor 6 years ago should be considered as follicular carcinoma, as a mass developed in the left hip 6 years later and proved to be thyroid tissue even though the primary thyroid tumor presented benign histology.

Thyroid follicular carcinoma with bone metastasis required multimodal and comprehensive treatment, including radioactive iodine therapy, palliative treatment, surgical treatment and targeted drug therapy. Among them, radioactive iodine therapy, as the first-line treatment, has good efficacy. FTC is the most sensitive to ^131^I, and most of the metastatic lesions retain the iodine uptake function. Therefore, ^131^I therapy is an effective means to treat residual primary lesions and metastatic lesions of FTC. Woodrum et al. believed that ^131^I treatment was one of the comprehensive treatment methods of FTC, which can treat recurrent or metastatic cancer (9). In the case of single distant metastasis, surgical resection of the metastasis and postoperative ^131^I treatment can achieve good therapeutic effect (10–12). In the case of multiple metastases, ^131^I treatment should be the first choice, which can effectively reduce serum Tg level, relieve pain and improve patients’ quality of life. Lee et al (13) believed that the survival rate of patients with bone metastasis was significantly lower than that of patients with lung metastasis and lymph node metastasis, mainly due to poor uptake of ^131^I in bone metastasis. The general principle of ^131^I treatment was to perform total or subtotal thyroidectomy to enhance iodine concentration in metastatic carcinoma before ^131^I therapy was performed. But if the size of the metastasis was too large, the efficacy of ^131^I therapy was limited, and other treatments should be combined. Palliative care mainly included vascular embolization, radiofrequency ablation and external irradiation, which can significantly relieve pain and nerve compression symptoms and improve patients’ quality of life. Surgical treatment was mainly suitable for persistent pain at bone metastasis or poor medical treatment effect. Anlotinib was a novel small-molecule multi-target tyrosine kinase inhibitor, which can strongly inhibit vascular endothelial growth factor receptor, platelet-derived growth factor receptor, fibroblasts growth factor receptor, stem cell factor receptor and other targets, and had anti-tumor angiogenesis and tumor growth inhibition effects (14, 15). Sun (16) et al. conducted a phase II study of anlotinib in the treatment of patients with locally advanced or metastatic thyroid cancer, in which patients with unresectable locally advanced or metastatic thyroid cancer were given oral anlotinib treatment until disease progression, death or unacceptable toxic reactions happened. the primary endpoint being progression-free survival. Results: a total of 58 patients enrolled did not meet the primary endpoint of progression-free survival at the time of analysis, 56.9% had a partial response, and progression-free survival at 48 weeks was 85.5%.The safety profile of anlotinib was available in a phase IIB study involving 62 patients with locally advanced or metastatic thyroid cancer (17). These results suggested that anlotinib shows persistent antitumor activity with controllable adverse reactions in locally advanced or metastatic thyroid cancer, which may promise a new effective treatment for patients with advanced or metastatic thyroid cancer. In this case, after bone metastasis of thyroid follicular carcinoma was pathologically confirmed by biopsy of the left pelvis, residual thyroid lobule resection was performed and ^131^I was combined to further remove the lesion. One noteworthy feature of the case was that the volume of metastatic lesions was huge at the first diagnosis of the patient, which was difficult to be cured by iodine therapy. Therefore, multimodal combined treatment was required. Arterial embolization therapy + ultrason-guided radiofrequency ablation were conducted for the damaged lesions in the left pelvic metastatic foci, followed by targeted therapy + pelvic radiotherapy, finally inactivating all the huge metastatic foci, thus successfully controlling the huge pelvic tumor. This case provided a reference method for the treatment of difficult large metastases. For large metastases, the combination of multiple methods can safely and effectively inactivate the tumor body. Cooperating with them, radiotherapy and targeted drugs could be used to control the progression of residual tumors. Such treatment was a relatively practical and effective method in that it not only achieve reliable effect, but can also reduce the side effects caused by high dose of targeted drugs and correspondingly help prolong the survival and improve the quality of life of patients.

Based on this case and related studies, conclusion could be made that the diagnosis of FTC was difficult and yet extremely important. For FTC with distant metastasis, relevant examinations should be improved before surgery, the patient’s general condition should be comprehensively evaluated, and multidisciplinary experts should combine efforts to formulate individual treatment plan peculiar to the patient’s situation, which can reduce postoperative complications, improve the quality of life and prolong the survival period.

## Data availability statement

The original contributions presented in the study are included in the article/supplementary material. Further inquiries can be directed to the corresponding author.

## Ethics statement

Written informed consent was obtained from the participant/patient(s) for the publication of this case report.

## Author contributions

All authors listed have made a substantial, direct and intellectual contribution to the work, and approved it for publication.

## References

[1] Kim HK Shin HJ Hahn SY Lyun OY Kim SW Parket KW. Prediction of follicular thyroid carcinoma associated with distant metastasis in the preoperative and postoperative model. Head Neck (2019) 41(8):2507–13. doi: 10.1002/hed.25721 30891875

[2] Bongiovanni M Sykiotis GP Rosa SL Bisig B Trimech M Missiaglia E. Macrofollicular variant of follicular thyroid carcinoma: a rare underappreciated pitfall in the diagnosis of thyroid carcinoma. Thyroid (2020) 30(1):72–80. doi: 10.1089/thy.2018.0607 31701808

[3] de Melo TG Zantut-Wittmann DE Ficher E da Assumpção LVM. Factors related to mortality in patients with papillary and follicular thyroid cancer in long-term follow-up. J Endocrinol Invest (2014) 37(12):1195–200. doi: 10.1007/s40618-014-0131-4 25037473

[4] Yu Y Bovenhuis H Zhou W Laport K AM Groenen M PMA Crooijmans R. Deleterious mutations in the TPO gene associated with familial thyroid follicular cell carcinoma in Dutch German longhaired pointers. Genes (Basel) (2021) 12(7):997. doi: 10.3390/genes12070997 34209805 PMC8306087

[5] Parameswaran R Hu JS En NM Tan WB Yuan NK. Patterns of metastasis in follicular thyroid carcinoma and the difference between early and delayed presentation. Ann R Coll Surg Engl (2017) 99(2):151–4. doi: 10.1308/rcsann.2016.0300 PMC5392828 27659362

[6] McHenry CR Phitayakorn R. Follicular adenoma and carcinoma of the thyroid gland. Oncologist (2011) 16(5):585–93. doi: 10.1634/theoncologist.2010-0405 PMC3228182 21482585

[7] Agarwal S Rao S Arya A Gupta K Arora R Dhawan I. Follicular thyroid carcinoma with metastasis to skin diagnosed by fine needle aspiration cytology. Indian J Pathol Microbiol (2008) 51(3):430–1. doi: 10.4103/0377-4929.42552 18723982

[8] Meadows KM Amdur RJ Morris CG Villaret DB Mazzaferri EL Mendenhall WM. External beam radiotherapy for differentiated thyroid cancer. Am J Otolaryngol (2006) 27(1):24–8. doi: 10.1016/j.amjoto.2005.05.017 16360819

[9] Woodrum DT Gauger PG. Role of 131I in the treatment of well differentiated thyroid cancer. J Surg Oncol (2005) 89(3):114–21. doi: 10.1002/jso.20185 15719384

[10] Xiong YX Zhang X. Observation on the effects of radioactive iodine treatment in bone metastasis from differentiated thyroid cancer. Jilin Med J (2008) 51(11):904–6.

[11] Paschke R Lincke T Müller SP Kreissl MC Dralle H Fassnacht M. The treatment of well-differentiated thyroid carcinoma. Dtsch Arztebl Int (2015) 112(26):452–8. doi: 10.3238/arztebl.2015.0452 PMC4515787 26205749

[12] Aamna H Madeeha K Saima Riaz M Khalid N Humayun B. Follicular thyroid carcinoma: disease response evaluation using American thyroid association risk assessment guidelines. Eur Thyroid J (2015) 4:260–5. doi: 10.1159/000442237 PMC4716413 26835430

[13] Lee J Soh EY. Differentiated thyroid carcinoma presenting with distant metastasis at initial diagnosis clinical outcomes and prognostic factors. Ann Surg (2010) 251(1):114–9. doi: 10.1097/SLA.0b013e3181b7faf6 19779325

[14] Xie CY Wan XZ Quan HT Zheng MY Fu L Li Y. Preclinical characterization of anlotinib, a highly potent and selective vascular endothelial growth factor receptor-2 inhibitor. Cancer Sci (2018) 109(4):1207–19. doi: 10.1111/cas.13536 PMC5891194 29446853

[15] Taurin S Yang CH Reyes M Cho SP Coombs DM Jarboe EA. Endometrial cancers harboring mutated fibroblast growth factor receptor 2 protein are successfully treated with a new small tyrosine kinase inhibitor in an orthotopic mouse model. Int J Gynecol Canc (2018) 28(1):152–60. doi: 10.1097/IGC.0000000000001129 PMC5735020 28953502

[16] Sun YK Du F Gao M Ji QH Li ZD Zhang Y. Anlotinib for the treatment of patients with locally advanced or metastatic medullary thyroid cancer. Thyroid (2018) 28(11):1455–61. doi: 10.1089/thy.2018.0022 30142994

[17] Xiao YJ Wei LL Gui X Lv CY Ding HL Lu CL. Efficacy and safety of targeted therapeutics for patients with radioiodine-refractory differentiated thyroid cancer:Systematic review and network meta-analysis. Front Pharmacol (2022) 13:933648. doi: 10.3389/fphar.2022.933648 36091770 PMC9461142

